# Self-Reporting Conjugated Polymer Nanoparticles for Superoxide Generation and Detection

**DOI:** 10.1021/acsami.4c06749

**Published:** 2024-07-15

**Authors:** Anna L. Clayborn, Jaclyn A. Rebstock, Lauren J. Camardella, Elizabeth P. Comeau, Sonali K. Dabhi, Eleanor G. Graber, Thomas H. Joyce, Isabelle N. Maricar, Brianna N. Pinckney, Devika Puri, Tayli B. Shekleton, Quyen Beatrice T. Tran, Elizabeth J. Harbron

**Affiliations:** Department of Chemistry, William & Mary, Williamsburg, Virginia 23187-8795, United States

**Keywords:** conjugated polymer nanoparticles, semiconducting polymer dots, superoxide, fluorescence, energy transfer, electron transfer

## Abstract

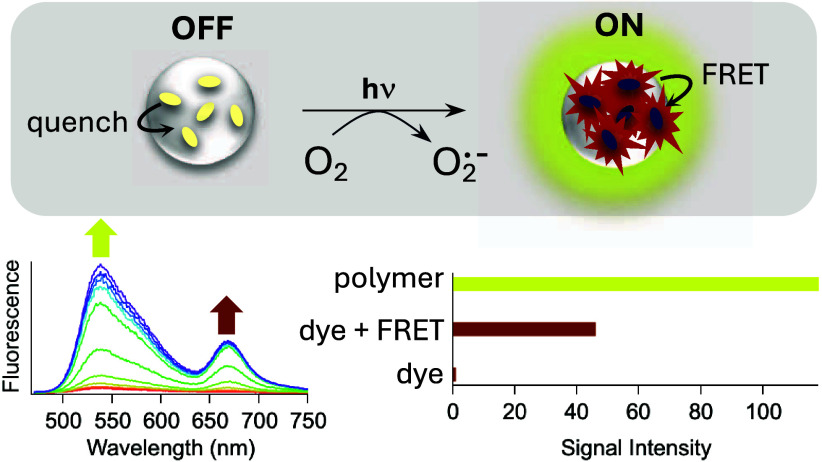

Conjugated polymer nanoparticles (CPNs or Pdots) have become increasingly popular fluorophores for multimodal applications that combine imaging with phototherapeutic effects. Reports of CPNs in photodynamic therapy applications typically focus on their ability to generate singlet oxygen. Alternatively, CPN excited states can interact with oxygen to form superoxide radical anion and a CPN-based hole polaron, both of which can have deleterious effects on fluorescence properties. Here, we demonstrate that CPNs prepared from the common conjugated polymer poly[(9,9-dioctylfluorenyl-2,7-diyl)-*alt*-co-(1,4-benzo-{2,1′,3}-thiadiazole)] (PFBT, also known as F8BT) generate superoxide upon irradiation. We use the same CPNs to detect superoxide by doping them with a superoxide-responsive hydrocyanine dye developed by Murthy and co-workers. Superoxide induces off-to-on fluorescence switching by converting quenching hydrocyanine dyes to fluorescent cyanine dyes that act as fluorescence resonance energy transfer (FRET) acceptors for PFBT chromophores. Amplified FRET from the multichromophoric CPNs yields fluorescence signal intensities that are nearly 50 times greater than when the dye is excited directly or over 100 times greater when signal readout is from the CPN channel. The dye loading level governs the maximum amount of superoxide that induces a change in fluorescence properties and also influences the rate of superoxide generation by furnishing competitive excited state deactivation pathways. These results suggest that CPNs can be used to deliver superoxide in applications in which it is desirable and provide a caution for fluorescence-based CPN applications in which superoxide can damage fluorophores.

## Introduction

Light’s power to image, sense, and deliver therapeutic treatment in biological systems has driven the development of functional fluorophores that operate in aqueous environments. Conjugated polymer nanoparticles (CPNs or Pdots) possess the light-harvesting power of their parent hydrophobic conjugated polymers but are stably suspended in water due to their negatively charged surface.^1^ CPNs’ outstanding extinction coefficients and good fluorescence quantum yields result in brighter fluorescence than comparable organic dyes and quantum dots.^2^ The photostability of CPNs likewise exceeds that of common fluorophores.^3^ The combination of exceptional photophysical properties with aqueous compatibility has made CPNs compelling fluorophores for biomedical applications from imaging to theranostics.^4−7^ Here, we present CPNs that generate superoxide radical anion and act as a selective fluorescent reporter of superoxide in water.

CPNs’ remarkable ability to absorb light makes them well-suited to multimodal applications that combine fluorescence with other excited state deactivation pathways to produce phototherapeutic effects.^8−10^ In particular, the presence of oxygen creates additional reactive pathways for CPN excited states that can lead to the generation of reactive oxygen species (ROS). It has long been known that CPNs doped or covalently functionalized with photosensitizers can generate ROS in high yield for photodynamic therapy (PDT) applications.^11−14^ More recently, some all-organic CPNs have been shown to generate ROS upon irradiation in the absence of an additional photosensitizer.^15−18^ These studies have focused primarily on Type II photosensitization in which CPNs generate singlet oxygen (^1^O_2_) via energy transfer from the CPN triplet state (T_1_). Alternatively, CPNs in the T_1_ state can donate an electron to oxygen to form the superoxide radical anion (O_2_^•–^) and a CPN-based hole polaron as products. Known as Type I photosensitization, this pathway can be a desirable addition to PDT applications in hypoxic environments because superoxide generation consumes less oxygen than singlet oxygen generation.^19,20^ However, the same reaction is undesirable in many fluorescence-based applications because photogenerated hole polarons act as fluorescence quenchers, reducing CPN fluorescence intensity.^21−23^ Hole polarons are also implicated in the photodegradation mechanisms of some conjugated polymers.^24^

We developed an interest in superoxide generation by CPNs after observing that some organic dyes photobleached to a greater extent when doped onto CPNs than when studied in homogeneous solution. Superoxide is a well-known contributor to the photocatalytic degradation of organic dyes,^25^ and ROS generation has been identified as a treatment method for textile dye effluent.^26^ Indeed, conjugated polymer nanostructures were shown to degrade over 90% of rhodamine B and methylene blue dyes in water upon irradiation.^27^ Careful study with various ROS scavengers demonstrated that superoxide was the dominant species involved in photocatalytic dye degradation by the conjugated polymer structures. Superoxide has also been specifically implicated in the photobleaching of poly(*p*-phenylenevinylene)-based conjugated polymers.^28^ The reaction of conjugated polymer donors with oxygen acceptors to produce superoxide radical anion has been studied in thin films,^29^ devices with charge transport layers,^30^ and nanofibers.^31^ Superoxide generation by CPNs has been demonstrated in tellurophene-containing CPNs in which intersystem crossing to the triplet state - and, hence, ROS production - is enhanced by the heavy atom effect.^32^ CPNs have also been used as a chemiluminescence-based reporter of superoxide.^33^ Despite its importance for PDT and fluorescence-based applications, superoxide generation by common, all-organic CPNs has not been intensively studied.

**Scheme 1:**
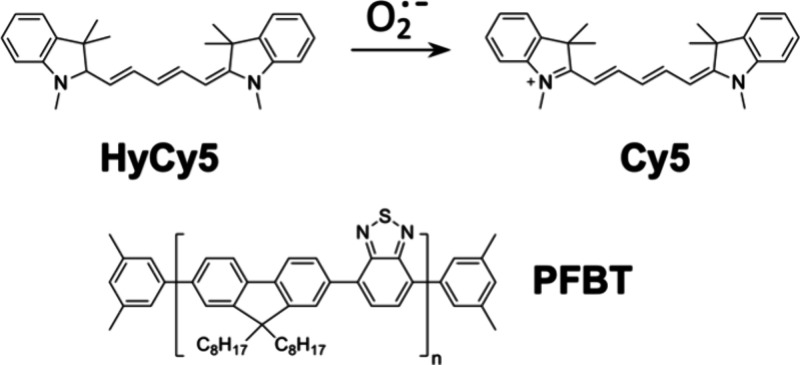
Chemical Structures of Conjugated Polymer PFBT and the Reduced (HyCy5) and Oxidized (Cy5) Forms of Dye Dopants

Our goals are to demonstrate that CPNs generate superoxide upon irradiation in aqueous environments and to take advantage of CPNs’ light-harvesting properties to detect superoxide with amplified signal intensity. Toward these ends, we developed dye-doped CPNs that both generate and report on superoxide. CPNs are prepared from the commonly used conjugated polymer poly[(9,9-dioctylfluorenyl-2,7-diyl)-*alt*-co-(1,4-benzo-{2,1′,3}-thiadiazole)] (PFBT, also known as F8BT, Scheme 1). These CPNs are doped with HyCy5,^34,35^ a known turn-on probe for superoxide that transforms to fluorescent Cy5 upon reaction with superoxide (Scheme 1). HyCy5 is an efficient quencher of PFBT fluorescence while Cy5 exhibits sensitized emission via fluorescence resonance energy transfer (FRET) from the conjugated polymer chromophores. The HyCy5-doped CPNs thus serve as an off-to-on fluorescence probe for superoxide. Here, we detect CPN-generated superoxide by the activation of fluorescence and separately by a Cytochrome C assay. All of the PFBT CPNs studied here generate superoxide, which is a concern for fluorescence-based applications in which this reaction is deleterious. The fluorescence-based superoxide detection reported here is enhanced by the CPNs’ exceptional ability to deliver excitation energy to FRET-accepting dyes, an effect known as amplified energy transfer.^36^ In the HyCy5-doped CPNs, the probe’s fluorescence intensity is amplified by a factor of nearly 50. When the brighter polymer emission is used for fluorescence readout, this amplification increases to a factor of over 100. The combination of off-to-on probe design and signal amplification makes the dye-doped CPNs a promising platform for superoxide detection.

## Experimental Section

### Materials

All chemicals were obtained from Fisher or Sigma-Aldrich and used as received unless otherwise specified. Poly[(9,9-dioctylfluorenyl-2,7-diyl)-*co*-1,4-benzo-{2,1′-3}-thiadiazole] (PFBT) with an average molecular weight of 83,000 and polydispersity of 3.8 was obtained from Montreal Optoelectronics (Quebec, Canada). The dye 2-[5-(1,3-dihydro-1,3,3-trimethyl-2*H*-indol-2-ylidene)-1,3-pentadienyl]-1,3,3-trimethyl-3*H*-indolium iodide (HIDC iodide) was obtained from Exciton.

### Nanoparticle Preparation

CPNs were prepared by a literature procedure.^37^ A stock solution of conjugated polymer PFBT (1 mg/mL) in anhydrous THF was stirred under argon for at least 4 h before use. A precursor solution (0.04 mg/mL) was prepared by diluting the PFBT stock with THF. The precursor solution was filtered through a 0.7 μm glass fiber filter and then sonicated for 30 s to ensure homogeneity. A 1 mL portion of this solution was injected into 8 mL of sonicating ultrapure water, which was then sonicated for an additional 2 min. Doubly concentrated CPNs used in the Cytochrome C assay were prepared by injecting a 2 mL portion of the precursor solution into the sonicating ultrapure water and following the same procedure. THF was removed via heating (50 °C) and argon bubbling for 30 min, and the aqueous suspension of CPNs was then filtered through a 0.7 μm filter layered over a 0.22 μm filter. A Nicomp 380 ZLS was used to determine nanoparticle size distributions and zeta potentials via dynamic and electrophoretic light scattering, respectively.

### Spectroscopy and Photochemistry

Absorption and fluorescence measurements were performed on an Agilent Technologies Cary 60 UV–vis spectrophotometer and a Varian Eclipse fluorimeter, respectively. CPNs were studied in aqueous suspension in semimicro quartz cuvettes (Starna, 10 mm × 4 mm interior dimensions). Visible irradiation (455 or 625 nm) was provided by a four-wavelength high power LED Source (ThorLabs, DC4100). Irradiation was delivered to the top of the sample cuvette in the absorbance and fluorescence instruments by a liquid light guide (ThorLabs, LLG0538). Irradiation intensity values at 455 nm were determined using a Coherent FieldMate Laser Power Meter.

### Hydrocyanine-Doped CPN Fluorescence Assay

HyCy5 was prepared from HIDC iodide according to literature procedure.^35^ HyCy5 was doped onto the CPN surface by adding a small amount of a HyCy5 stock solution in methanol to the CPN suspension, which was then manually agitated. The volume of HyCy5 stock solution added was adjusted for each preparation so that the concentration of HyCy5 dyes in CPNs would be 2.5–25 wt % relative to PFBT. Suspensions of doped CPNs were tested for dye leaching by spinning in a centrifugal filtration device (Amicon Ultra-4 centrifugal filter with a molecular weight cutoff of 100 000) in accordance with a literature procedure.^38^ Fluorescence spectra were recorded after sequential irradiation intervals (5 s). Fluorescence kinetic trajectories were collected at 537 and 671 nm emission wavelengths during continuous LED irradiation. The excitation wavelength was 450 nm for all assays except the amplification assay, where it alternated between 450 and 625 nm. Samples for degassed experiments were purged with argon for 90 min prior to study.

### Methylene Blue Assay

Methylene blue stock solution (40 μL of a 0.01 v/v solution) was injected into a 600 μL sample of HyCy5-doped CPNs. Identical samples were irradiated with either 455 or 625 nm light for 35 s. To determine the experimental parameters required for methylene blue’s production of singlet oxygen in water, the change in the absorbance of 1,3-diphenylisobezofuran was monitored during irradiation of methylene blue with 625 nm light. To get hydrophobic 1,3-diphenylisobezofuran into an aqueous solution, 1,3-diphenylisobezofuran stock (4 mM) was injected into sodium dodecyl sulfate micelles, and methylene blue stock (20 μL) was then added to the micelles (320 μL). Absorbance spectra were recorded at 5 s intervals during irradiation with 625 nm light.

### Cytochrome C Reduction

Poly-l-lysine hydrobromide (PLL) stock solution (1 mL, 0.22 mg/mL) was injected into a doubly concentrated CPN suspension and allowed to stir under argon for at least 5 h. The PLL-coated CPNs were diluted with ultrapure water and 1x phosphate buffered saline (150 μL) to achieve 300 μL sample aliquots with a constant CPN concentration. Cytochrome C stock (4 μL, 1 mM) was added to the diluted CPN aliquot and incubated for 20 min. If used, 4 μL of superoxide dismutase (SOD) stock (60 units/mL) was added to the sample immediately prior to its introduction to the cuvette. Sample absorption at 550 nm was recorded during a 2 min period of 455 nm LED irradiation of variable intensity.

## Results and Discussion

### Probe Design

The components of dye-doped CPNs that both generate and detect superoxide are depicted in Scheme 1. We selected PFBT as the conjugated polymer due to its outstanding fluorescence properties and its prior use in CPN-based photodynamic therapy applications. PFBT-derived CPNs exhibit bright fluorescence^2^ and are more photostable than some other CPNs.^3,24^ These properties make PFBT an excellent fluorescence resonance energy transfer (FRET) donor to doped dyes, as we^39−42^ and others^18,43,44^ have demonstrated. CPNs prepared from PFBT or closely related polymers have been also doped with photosensitizers to generate singlet oxygen for photodynamic therapy via FRET.^11,12^

**Figure 1:**
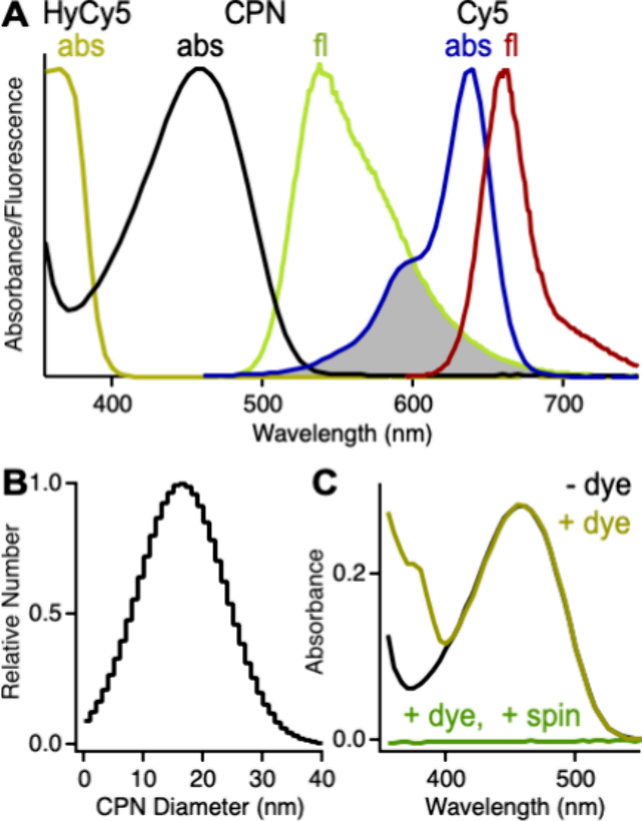
(A) Absorbance of HyCy5 (tan), absorbance (black) and fluorescence (yellow-green) of PFBT CPNs, and absorbance (blue) and fluorescence (red) of Cy5 dyes with shading depicting donor–acceptor spectral overlap for FRET. (B) Size distribution of PFBT CPNs measured in aqueous suspension by dynamic light scattering. (C) Absorbance of PFBT CPNs before (black) and after (tan) doping with HyCy5 and after the doped sample was spun in a centrifugal filtration device (green).

For superoxide detection, we sought to pair PFBT CPNs with a suitable dye that would become emissive and act as a FRET acceptor upon reaction with superoxide. Out of the many dyes that detect superoxide via fluorescence or chemiluminescence,^45−47^ we selected HyCy5, a hydrocyanine dye developed by Murthy and co-workers,^34,35^ for its selectivity, ease of preparation, and FRET-compatible fluorescence properties in its Cy5 form (Scheme 1). HyCy5 dyes are nonfluorescent until they react with superoxide, which oxidizes them to fluorescent Cy5 via a multistep radical mechanism that may occur via electron transfer and proton transfer steps or hydrogen atom transfer followed by electron transfer.^48^ Murthy showed that the hydrocyanines respond selectively to superoxide and hydroxyl radical over singlet oxygen and other ROS, making them ideal for this work.^34,35^ FRET from the CPNs to the oxidized form of the dye is expected to be favored due to the spectral overlap between the donor PFBT fluorescence and the acceptor Cy5 absorbance (Figure 1A). Algar previously studied energy transfer in Cy5-doped PFBT CPNs and concluded that FRET is the dominant mechanism.^43^ No energy transfer is expected to occur between PFBT and HyC5 as the reduced form of the dye absorbs only in the UV and very slightly in the blue region of the spectrum and has no spectral overlap with PFBT fluorescence (Figure 1A).

Prepared by a nanoprecipitation procedure adapted from McNeill et al.,^37^ the CPNs have an average diameter of 16 ± 7 nm (Figure 1B). Hydrophobic HyCy5 dyes are doped onto the CPN surface via addition of a small amount of methanol stock solution to the aqueous suspension of CPNs. To test whether the dyes are stably adsorbed onto the CPN surface, we spun a doped CPN suspension in a centrifugal filtration device that separates the CPNs from the aqueous medium (see Experimental Section). No evidence of HyCy5 dyes can be seen in the absorbance spectrum of the aqueous filtrate, demonstrating that the dyes are stably doped onto the CPNs (Figure 1C).

**Figure 2:**
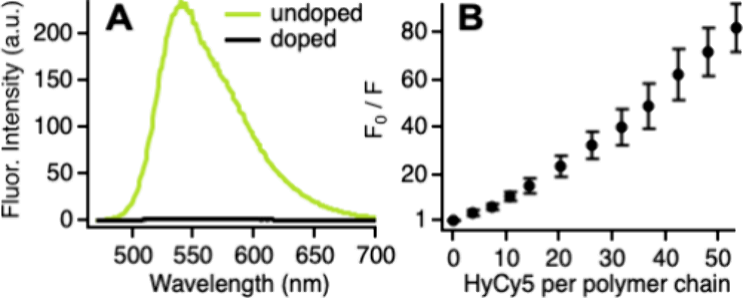
(A) Fluorescence spectra of undoped (yellow-green) and HyCy5-doped (black) CPNs. (B) Stern–Volmer plot showing quenching of CPN fluorescence by HyCy5 dyes. Values are the average of 5 runs with error bars representing the standard deviation.

We designed HyCy5-doped CPNs to be a ratiometric superoxide reporter. We originally expected to observe unquenched green-yellow fluorescence from PFBT due to the absence of a FRET acceptor in as-prepared samples. The fluorescence spectra would exhibit anticorrelated changes as superoxide is formed, with emission intensity decreasing for the donor PFBT while increasing for the acceptor Cy5 as it is generated. Instead, we observed that HyCy5 is an efficient quencher of PFBT fluorescence, producing CPNs that are virtually nonemissive in their as-prepared form (Figure 2A). The HyCy5 structure includes an aromatic amine that can serve as an electron donor. Quenching of other conjugated polymers by electron transfer from aromatic amines has been observed previously.^49,50^ In one related system, a porous conjugated polymer that contains benzothiadiazole subunits has also been shown to be quenched by aromatic amines via photoinduced electron transfer.^50^

We further investigated the quenching of PFBT CPNs by HyCy5 by performing a Stern Volmer analysis (Figure 2B). The relationship between the extent of fluorescence quenching and the quencher concentration is given by F_0_/F = 1 + K_SV_[Q], where F_0_ and F are the CPN fluorescence intensities in the absence and presence of HyCy5, K_SV_ is the Stern Volmer constant, and [Q] is the molar concentration of the quencher.^51^ Here, we express the quencher concentration as the number of dyes per conjugated polymer chain. The Stern Volmer plot exhibits upward curvature at low dye loadings before becoming linear at ca. 20 dyes per polymer chain. This shape is reproducible and likely reflects the Poissonian distribution of quenchers at low loadings. In studies of other dye-doped CPNs, McNeill et al. have shown that K_SV_ represents the average number of polymer chains quenched per dye molecule when the data are plotted as a function quencher:donor molecular ratio.^52^ A fit of the linear portion of Figure 2B yields a K_sv_ of 1.9, indicating that each dye quenches ca. 2 polymer chains on average. While this value represents significant quenching, it is not nearly as large as that observed for some other dye-doped CPNs.^52^ The polydispersity of the PFBT used to prepare our CPNs introduces some uncertainty into this analysis, but we can draw the qualitative conclusion that the HyCy5-doped CPNs do not exhibit the extraordinary quenching demonstrated by some dye-doped CPNs.^52^ HyCy5 does not completely quench all fluorophores, which leaves some CPN excited states available for superoxide generation. We demonstrate further below that CPN quenching by HyCy5 affects the superoxide generation rate but does not prevent superoxide formation.

**Figure 3:**
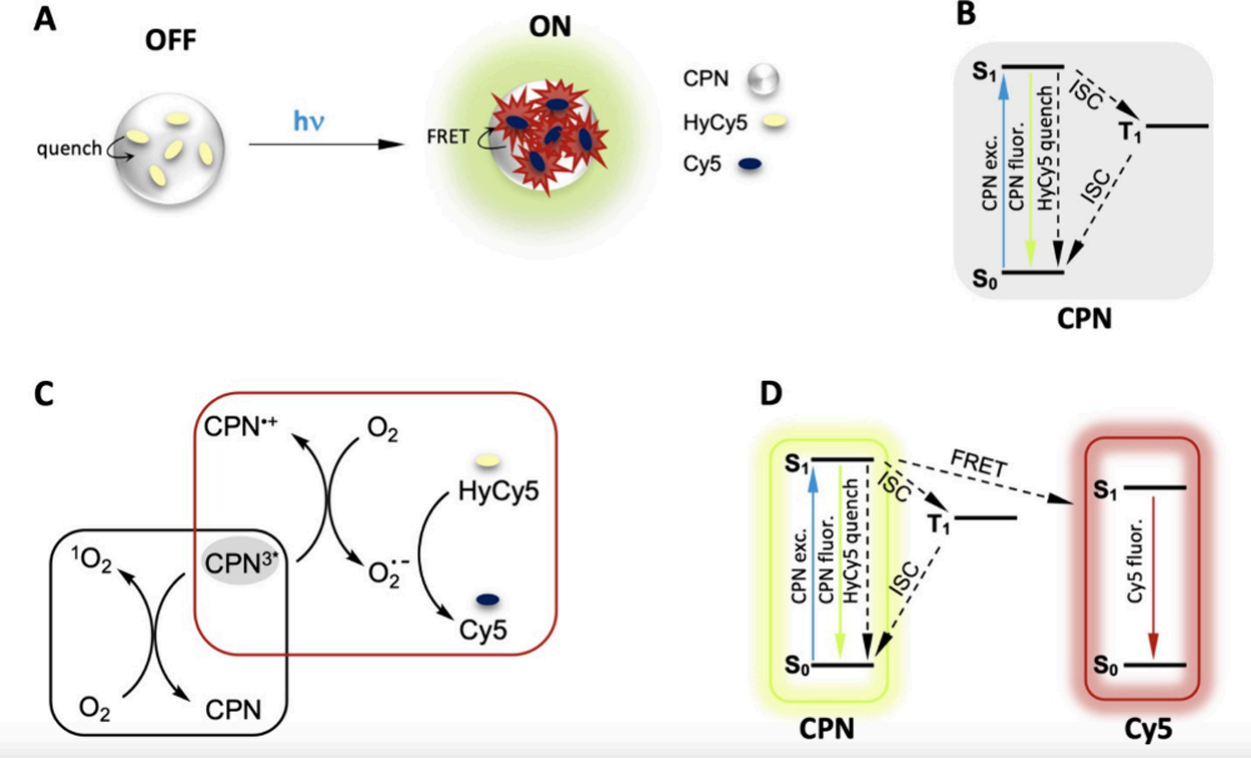
(A) Scheme for superoxide reporting by turn-on fluorescence in doped CPNs. (B) Jablonski diagram depicting key photophysical pathways in as-prepared HyCy5-doped CPNs. (C) Scheme for interaction of oxygen with CPN excited state. (D) Jablonski diagram depicting key photophysical pathways in doped CPNs after some HyCy5 dyes have been converted to Cy5. Internal conversion and other minor pathways are omitted for clarity in the Jablonski diagrams.

The ability of HyCy5 to act as an electron donor is lost when superoxide converts the dye to Cy5, where the aromatic amine carries a delocalized positive charge (Scheme 1). The HyCy5 quenching of CPN fluorescence thus presents the exciting opportunity to develop a turn-on fluorescence probe for superoxide (Figure 3A). Probes that feature a fluorescent turn-on response are generally preferred over those with a turn-off response due to their zero-background nature.^53^ In the present dye-doped nanoparticles, CPN fluorescence begins in a highly quenched state because HyCy5 quenching is the dominant pathway for deactivation of the S_1_ excited state (Figure 3B). Electron transfer from conjugated polymer excited states has long been thought to occur from the triplet state^54^ but is sometimes depicted as occurring from S_1_.^11^ We cannot exclude the possibility of electron transfer to oxygen from S_1_ but will depict this process as occurring out of T_1_ in alignment with much of the literature.^17,55^ PFBT has a low intersystem crossing quantum yield of 0.019^56^ (polymer film), but higher intensity LED illumination is expected to sufficiently populate the T_1_ state to activate superoxide production. Electron transfer from the excited CPN to molecular oxygen yields superoxide, which in turn converts quenching HyCy5 dyes to fluorescent Cy5 dyes (Figure 3C). This reaction also creates a CPN hole polaron (CPN^•+^), which will be considered further below. CPN^3*^ can also transfer energy to molecular oxygen to form singlet oxygen, which we show further below does not contribute to the fluorescence response observed in our system. The superoxide-mediated conversion of HyCy5 to Cy5 relieves CPN fluorescence quenching while the presence of Cy5 activates the FRET pathway. The fluorescence output of this system will thus be a 2-color combination of fluorescence from the PFBT CPNs and the Cy5 dopants, with relative fluorescence intensities governed by FRET (Figure 3D).

### Superoxide Detection

**Figure 4:**
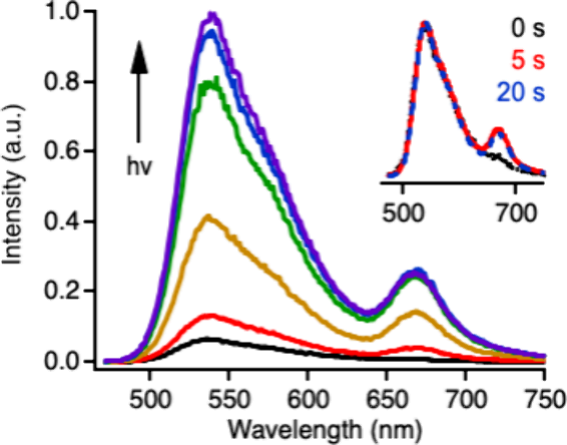
Fluorescence of 15 wt % HyCy5-doped CPNs before (black) and at 5 s intervals during irradiation (455 nm, 0.87 mW/cm^2^). Inset: normalized spectra recorded before irradiation and after 5 and 20 s of irradiation.

We stimulate superoxide production in CPNs by irradiating aqueous samples with a variable-intensity 455 nm LED. As shown in Figure 4, a fluorescence spectrum grows in upon irradiation and continues to increase in intensity during the irradiation period. The spectrum is dominated by the green-yellow fluorescence of PFBT with a smaller contribution from the red fluorescence of the FRET-excited Cy5 dyes in accordance with the Jablonski diagram (Figure 3D). We subjected an irradiated sample of doped CPNs to a centrifugal filtration test to determine whether any of the oxidized Cy5 dyes leach into the water after their formation. No Cy5 fluorescence was detected in the aqueous filtrate (not shown), indicating that the dyes remain stably adsorbed to the CPN surface following the HyCy5-to-Cy5 conversion. The dye-doped CPNs maintain their composition and meet the revised goal of exhibiting off-to-on fluorescence switching in response to superoxide.

The growth of the PFBT and Cy5 peaks appears to be highly correlated. Indeed, normalized spectra from early and late in the irradiation period are virtually indistinguishable (Figure 4 inset). CPNs doped with FRET-accepting fluorescent dyes typically show an anticorrelated decrease in CPN fluorescence and increase in dye fluorescence as the dye loading is increased.^43^ Here, each conversion of a quenching HyCy5 dye to a Cy5 dye removes a PFBT quencher from the system, increasing the population of PFBT excited states available for other deactivation pathways, including FRET. Given the multiple competing pathways that affect the PFBT donor intensity in this system, it is not possible to rigorously calculate the FRET efficiency from the Figure 4 spectra. We can estimate the proximity ratio, a relative form of FRET efficiency used in some single-molecule spectroscopy experiments, which depends on the fluorescence intensities of the donor and acceptor peaks upon donor excitation and is equal to F_Cy5_/(F_CPN_+F_Cy5_).^57^ This value is ca. 20% in our system, which is lower than one might expect given that FRET operates over a longer distance than electron transfer quenching.^58^ However, we show further below that concentration of Cy5 dyes produced in the experiment appears to be low and that some HyCy5 quenchers remain unreacted throughout the experiment. The observed spectra are likely the result of the competition between electron transfer involving many HyCy5 dyes, FRET involving a few Cy5 dyes, and other pathways.

**Figure 5:**
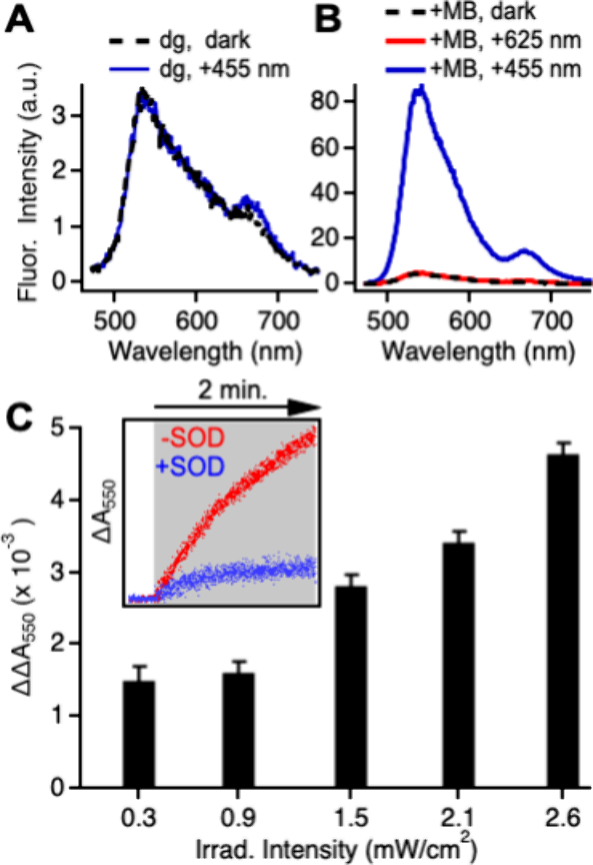
Fluorescence spectra of HyCy5-doped CPNs in aqueous suspension (A) under degassed conditions in the dark before (black) and after (blue) irradiation (40 s, 455 nm); (B) with methylene blue before (black) and after irradiation to produce singlet oxygen (red, 35 s, 625 nm) and after irradiation to produce a positive control (blue, 35 s, 455 nm). Fresh aliquots of the same sample were used for each irradiation exposure. (C) Difference in change of absorbance of Cyt C at 550 nm upon irradiation (2 min, 455 nm) in the presence and absence of SOD as a function of irradiation intensity. Results shown are the average of 3 measurements. Inset: representative traces of change of absorbance in presence and absence of SOD during irradiation (2 min, 455 nm, 2.6 mW/cm^2^).

We conducted a series of control experiments to verify that it is superoxide that induces the fluorescence activation shown in Figure 4. The first step in the superoxide-mediated oxidation of HyCy5 to Cy5 may be the loss of an electron from the amine.^34,48^ However, the same mechanistic step likely plays a role in the quenching of CPN fluorescence by photoinduced electron transfer from HyCy5. To determine whether the observed fluorescence activation is simply due to oxidation of HyCy5 by the CPNs, we degassed doped CPNs and studied the response of the sample to LED irradiation. The doped and degassed sample exhibits a highly quenched fluorescence spectrum, as expected (Figure 5A). The spectrum includes a minor contribution from already-oxidized Cy5, which forms very slowly over time in stored HyCy5 stock solutions. Upon LED irradiation, the only spectral change is a small increase in the intensity the Cy5 peak. This increase is not observed when the doped CPNs sit for a comparable amount of time in the dark before scanning and is also not observed when the dye is irradiated in solution in the absence of the CPNs. These results suggest that the observed response is due to a light-mediated interaction between the CPNs and HyCy5. However, the increase in fluorescence intensity observed here in the absence of oxygen (12%) is extremely small compared to that observed in the presence of oxygen (3,500% in the Figure 4 data). These results demonstrate that both light and oxygen are required to induce significant amounts of HyCy5 to Cy5 conversion and the resulting fluorescence activation.

We also investigated whether singlet oxygen contributes to the observed turn-on response, given that PFBT-derived CPNs are known to generate singlet oxygen upon irradiation.^18^ The HyCy5-to-Cy5 conversion was previously shown to be selective for superoxide and hydroxyl radical, which is formed from superoxide, over other ROS species.^34,35^ To generate singlet oxygen in the absence of superoxide, we used methylene blue, a well-known singlet oxygen photosensitizer.^59^ We established conditions that would produce singlet oxygen in water by irradiating methylene blue with a 625 nm LED in micelles in the presence of 1,3-diphenylisobenzofuran, a singlet oxygen reporter (not shown). We then irradiated methylene blue to generate singlet oxygen in the presence of HyCy5-doped CPNs, which do not absorb at 625 nm. Separately, we exposed a fresh aliquot of the same sample to the 455 nm irradiation used to produce the turn-on fluorescence response as a positive control. The HyCy5-doped CPNs did not show any response to singlet oxygen generated by methylene blue irradiation at 625 nm, while direct irradiation of the CPNs at 455 nm produced the familiar response (Figure 5B). Singlet oxygen does not contribute to the fluorescence turn-on response observed here.

Best practices for superoxide detection involve the use of multiple methods, since most individual methods carry limitations and weaknesses.^60^ Toward that end, we used a well-known spectroscopic method to verify that the CPNs are indeed producing superoxide upon irradiation. Cytochrome C (Cyt C) is reduced by superoxide, and the conversion of Cyt C–Fe^3+^ to Cyt C–Fe^2+^ can be monitored by tracking the increase in absorbance at 550 nm (ΔA_550_) as the reaction proceeds.^60,61^ The positively charged heme group in Cyt C is expected to bind electrostatically to the negatively charged CPN surface. Indeed, addition of Cyt C to aqueous suspensions of PFBT CPNs in the dark yielded an increase in A_550_ over time. We attributed this dark-state signal to redox chemistry between the CPNs and bound Cyt C. To eliminate these interactions and the resulting dark-state signal, we modified the CPN surface with poly(l-lysine) (PLL), a cationic polymer that has previously been shown^62^ to impart a positive surface charge to CPNs.^62−64^ Here, the zeta potential of PFBT CPNs switched from −24 mV to +28 mV following modification with PLL. When Cyt C is added to PLL-modified CPNs, the ΔA_550_ signal remains stable in the dark (Figure 5C inset at *t* < 0 min.).

Upon irradiation, CPNs can reduce Cyt C directly by photoinduced electron transfer or indirectly by generating superoxide, which then reduces Cyt C. To distinguish between these two pathways, we recorded the A_550_ signal in the absence and presence of superoxide dismutase (SOD), which scavenges superoxide and catalyzes its dismutation into oxygen and hydrogen peroxide.^65^ The difference in ΔA_550_ observed in the absence and presence of SOD (ΔΔA_550_) represents superoxide production. Representative traces (Figure 5C inset) show that reduction of Cyt C by superoxide dominates over direct reduction by the CPNs. As expected for a photoinduced process, the amount of superoxide detected increases with increasing irradiation intensity (Figure 5C). We can quantify superoxide production from ΔΔA_550_ values by using the difference in extinction coefficients between the reduced and oxidized forms of Cyt C.^61^ The concentration of superoxide detected in the Cyt C assay is in the 70–200 nM range, scaling with irradiation intensity.

**Figure 6:**
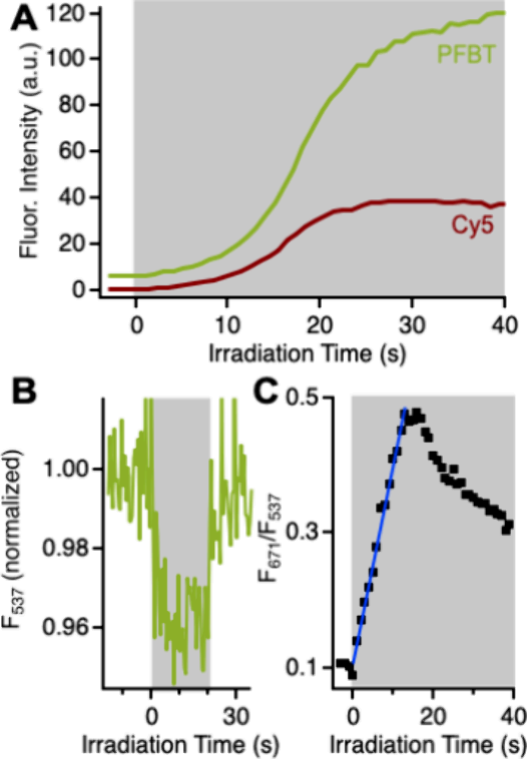
(A) Evolution of fluorescence intensity of 15 wt % doped CPNs during irradiation (shaded area, 455 nm, 0.87 mW/cm^2^) in PFBT (537 nm, yellow-green) and Cy5 (671 nm, red) channels. (B) PFBT Fluorescence intensity as a function of time in undoped CPNs before, during (shaded area, 455 nm, 0.87 mW/cm^2^), and after irradiation. (C) Ratio of fluorescence intensities from panel A as a function of irradiation time.

Having established that the dye-doped CPNs are indeed reporting on superoxide, we turned our focus to the fluorescence turn-on behavior of the system. Fluorescence kinetic data (Figure 6A) enable us to follow the evolution of the fluorescence signals during LED irradiation. We monitored the fluorescence λ_max_ values of the PFBT CPNs (537 nm) and of the Cy5 dyes doped onto the CPNs (671 nm), which we obtained from the difference of pre- and postirradiation spectra of the doped CPNs. Both the PFBT and Cy5 fluorescence signals are extremely stable before the LED is turned on at t = 0 s, indicating that superoxide is not being generated in detectable quantities. Presumably, the intensity of the excitation provided by the fluorimeter lamp is insufficient to overcome PFBT’s low intersystem crossing quantum yield and generate the T_1_ population required for detectable levels of superoxide formation. As soon as the higher intensity LED irradiation is applied, both PFBT and Cy5 fluorescence signals begin to grow in, slowly at early irradiation times (*t* < 10 s) followed by a more rapid increase (ca. 10–20 s). For reasons not yet understood, the PFBT signal continues to grow slightly after the Cy5 signal plateaus at later irradiation times. However, the stability of the Cy5 signal indicates that the dyes remain adhered to the CPN surface after the HyCy5 to Cy5 conversion, in agreement with the centrifugal filtration experiment described above.

The shape of the kinetic traces in Figure 6A reflect the interplay of photophysical processes at work in this system (Figure 3D). We previously observed similar S-shaped fluorescence kinetic traces while studying CPNs’ ability to sensitize the photochemical reactions of functional FRET-accepting dyes.^39,42^ Those cases and the present work all feature dyes that quench CPN fluorescence before a photochemical reaction or series of reactions converts them to a nonquenching form. Here, the HyCy5-to-PFBT molecular ratio is high enough that the HyCy5 quenching process dominates even after some dyes react with superoxide to form FRET-accepting Cy5 dyes. Only after some threshold number of dyes has reacted does the formation of additional Cy5 dyes have a more dramatic effect on the fluorescence intensity.

It is important to note that the reaction that produces superoxide also creates a CPN hole polaron (Figure 3C), a known fluorescence quencher. To assess the extent to which hole polaron formation might also influence the observed fluorescence intensities, we irradiated undoped nanoparticles and monitored the CPN fluorescence intensity over time (Figure 6B). Consistent with other bulk studies,^23^ the fluorescence intensity drops upon application of higher intensity irradiation due to hole polaron formation. Here, the fluorescence intensity decrease is to ca. 96% of initial intensity, and full recovery of fluorescence intensity occurs upon cessation of higher intensity irradiation. We looked for hole polaron recovery in experiments with doped CPNs by monitoring the postirradiation fluorescence intensity in numerous kinetic traces. Intensity jumps indicative of recovery from hole polaron quenching were observed in only a handful of traces, all of which had lower dye loadings than the standard loading used in this work (15 wt %). McNeill et al. previously showed that doping CPNs with nitrogen-containing small molecules suppresses hole polaron formation.^66^ It is possible that the HyCy5 dyes are acting in a similar capacity here.

The nonlinear growth of fluorescence intensity in this system could limit future quantitative applications in which a linear response would be needed for calibration. However, the correlated growth of fluorescence in the PFBT and Cy5 channels presents an opportunity to recover a linear signal. The ratio of Cy5 to PFBT fluorescence intensities (F_671_/F_537_) is indeed linear for the first 13 s of irradiation, corresponding to the periods of slow and moderate growth (Figure 6C). These results demonstrate the exciting possibility that HyCy5-doped CPNs could act as a ratiometric fluorescence reporter for superoxide.

### Amplification

**Figure 7:**
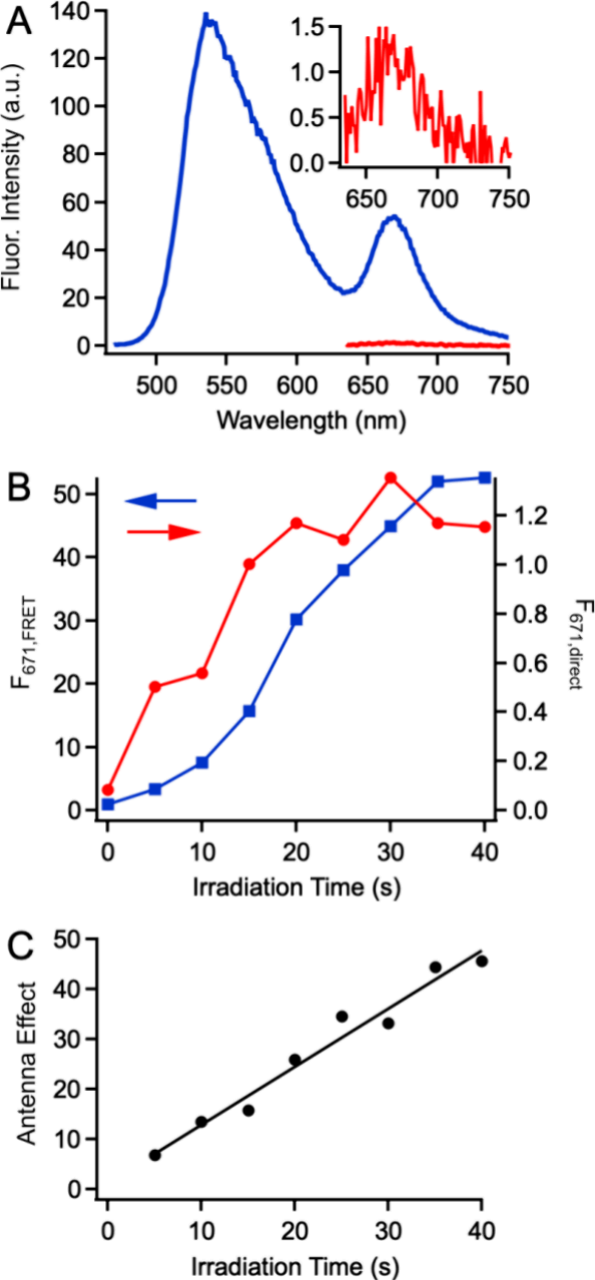
(A) Fluorescence spectra of 15 wt % dye-doped CPNs after irradiation (40 s, 455 nm, 0.87 mW/cm^2^) under FRET conditions (450 nm excitation, blue) and upon direct excitation of Cy5 dyes (625 nm, red). Inset: scaled version of the direct excitation spectrum. (B) Cy5 fluorescence intensity as a function of irradiation time upon FRET (blue) and direct (red) excitation. (C) Antenna effect as a function of irradiation time.

The fluorescence signals shown in Figure 6 are significantly more intense than they would be if HyCy5 dyes alone were used to probe superoxide. Indeed, amplification of the fluorescence signal is a key advantage of using HyCy5-doped CPNs as a fluorescence probe for superoxide as compared to the dyes alone. This amplification effect can be quantified by comparing the fluorescence intensity obtained when Cy5 is excited directly with that when it is excited via FRET from the CPNs. After 40 s of irradiation to produce the superoxide that converts HyCy5 dyes to Cy5, we recorded fluorescence spectra under direct and FRET excitation conditions (Figure 7A). The fluorescence intensity of Cy5 upon direct excitation is so low that the spectrum is barely visible without separate scaling. In contrast, Cy5 fluorescence is clearly seen upon FRET excitation due to the outstanding FRET donor capabilities of the multichromophoric CPNs.

We repeated the excitation experiment as a function of irradiation time to track the amplification during superoxide production (Figure 7B). The fluorescence signal for the directly excited dyes appears to grow fairly linearly before leveling off near the end of the irradiation period. In contrast, the signal for the FRET-excited dyes exhibits the now-familiar S-shaped curve that reflects the influence of quenching HyCy5 dyes, especially at early times. The fact that linearity can be restored by using the ratiometric signal negates a possible disadvantage of the FRET system.

Fluorescence amplification can be quantified by the antenna effect (AE), which is commonly defined as the ratio of the probe’s fluorescence intensity when excited via FRET to that when excited directly.^67^ Here, AE = (F_671,FRET_/F_671,direct_), where these terms represent the fluorescence intensity of the Cy5 peak under FRET (450 nm) and direct (625 nm) excitation conditions. AE for the HyCy5-doped CPNs increases linearly over the irradiation period, starting at 7 and reaching a maximum of 46 at the end of the experiment (Figure 7C). We previously examined AE over the course of a sensing experiment in dye-doped CPNs that exhibited an anticorrelated fluorescence response to mercury ions.^68^ In that work, AE remained relatively constant as increasing amounts of analyte were added over the course of the experiment. In the mercury-sensing system, the CPNs’ ability to act as a FRET donor remained constant throughout the experiment. Here, the degree to which PFBT chromophores in the CPNs can act as FRET donors in HyCy5-doped CPNs increases throughout the experiment as the number of quenching HyCy5 dyes decreases. The maximum AE observed here is similar to that observed in other systems we^41,68^ and others^44,69^ have studied in which CPNs amplify the emission of dye dopants via FRET. Since the fluorescence signal in the PFBT channel is correlated to that in the Cy5 channel, the PFBT signal can also be used as a fluorescence readout. In this form, AE becomes equal to F_537_/F_671,direct_, where F_537_ is the intensity of the PFBT fluorescence upon 450 nm excitation. For the experiment depicted in Figure 6, AE is 118 when the final F_537_ intensity is used as the amplified intensity. This means that the fluorescence intensity of the Cy5 dye is amplified over a hundredfold when the PFBT signal is used for fluorescence readout.

### Irradiation Intensity Dependence

Having demonstrated an off-to-on fluorescence response with signal amplification, we next sought to optimize the fluorescence response of HyCy5-doped CPNs. Our first goal was to maximize responsiveness while minimizing potential photobleaching effects. Our ideal fluorescence reporter will be photostable and will respond rapidly but not so much so that signal evolution cannot be studied. Toward this goal, we varied the rate of superoxide generation by manipulating the irradiation intensity. Since both the PFBT (F_537_) and Cy5 (F_671_) fluorescence signals increase as superoxide is generated, we used the larger F_537_ signal to track the time dependence of the fluorescent response in these optimization experiments.

**Figure 8:**
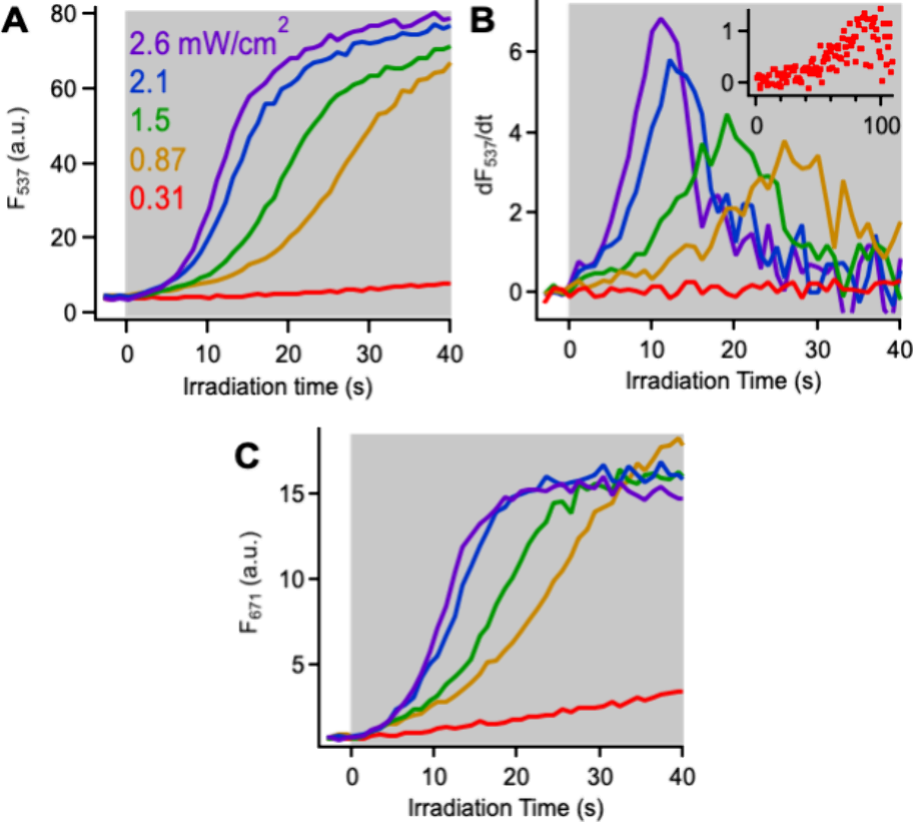
(A) Evolution of CPN fluorescence signal in 15 wt % doped CPNs during irradiation (shaded area) as a function of irradiation intensity. (B) Derivatives of the trajectories in (A) with inset depicting lowest-intensity data. (C) Evolution of Cy5 fluorescence signal during irradiation at same irradiation intensities as (A).

Increasing the irradiation intensity shifts the fluorescence response of the HyCy5-doped CPNs to earlier irradiation times (Figure 8A). The time at which the maximum increase in fluorescence intensity is observed shifts from 90 to 11 s as the irradiation intensity increases from 0.31 to 2.6 mW/cm^2^ (Figure 8B). These changes are consistent with the expectation that the rate of superoxide generation increases with increasing irradiation intensity, which we observed in the raw data from the Cyt C assay. The fluorescence signal’s temporal evolution can thus act as a marker of the rate of superoxide production. While the trace obtained at the lowest irradiation intensity responds too slowly to be of practical use, the remaining traces all demonstrate reasonable responsiveness. However, the doped Cy5 dyes are somewhat vulnerable to photobleaching. The fluorescence intensity of the Cy5 signal at t = 40 s decreases slightly and irregularly with increasing irradiation intensity (Figure 8C), indicating minor irreversible photobleaching. We thus determined that 0.87 mW/cm^2^ provided the best balance of good responsiveness with minimal photobleaching effects on the time scale of these experiments and employed these irradiation conditions for the remaining experiments.

Apart from the differences in time scale, the fluorescence responses to different irradiation intensities are quite similar. It appears that the F_537_ and F_671_ signals would reach the same end point if Cy5 photobleaching were not a factor (Figure 8A,C). Except for the lowest-intensity measurement, the area under the derivative curves is similar for all traces, increasing only 20% from 0.87 to 2.6 mW/cm^2^ (Figure 8B). Together, these results suggest that the HyCy5-doped CPNs are responding to similar superoxide amounts across the 0.87 to 2.6 mW/cm^2^ irradiation intensity range and thus exhibiting fluorescence responses of similar magnitude. This finding contrasts with the Cyt C assay, which showed that amount of superoxide produced by the CPNs scales with irradiation intensity as expected for a photogenerated species. It is also important to note that the fluorescence response in the doped CPNs reaches a plateau on a time scale when absorbance changes in the Cyt C assay are still being observed.

These observations point to two possible explanations. One is that the CPN dye loading level dictates the maximum amount of superoxide that can induce a change in the fluorescence intensity. A second explanation is that the presence of increasing numbers of Cy5 dyes as the experiment proceeds effectively shuts down superoxide production. FRET to superoxide-generated Cy5 dyes competes for the same CPN S_1_ excited state as the intersystem crossing that precedes superoxide formation (Figure 3D). The two explanations are not mutually exclusive, and the observed fluorescence behavior could represent some combination of the two. We next studied the influence of dye loading on the fluorescent response to investigate these possibilities.

### Dye Loading Dependence

**Figure 9:**
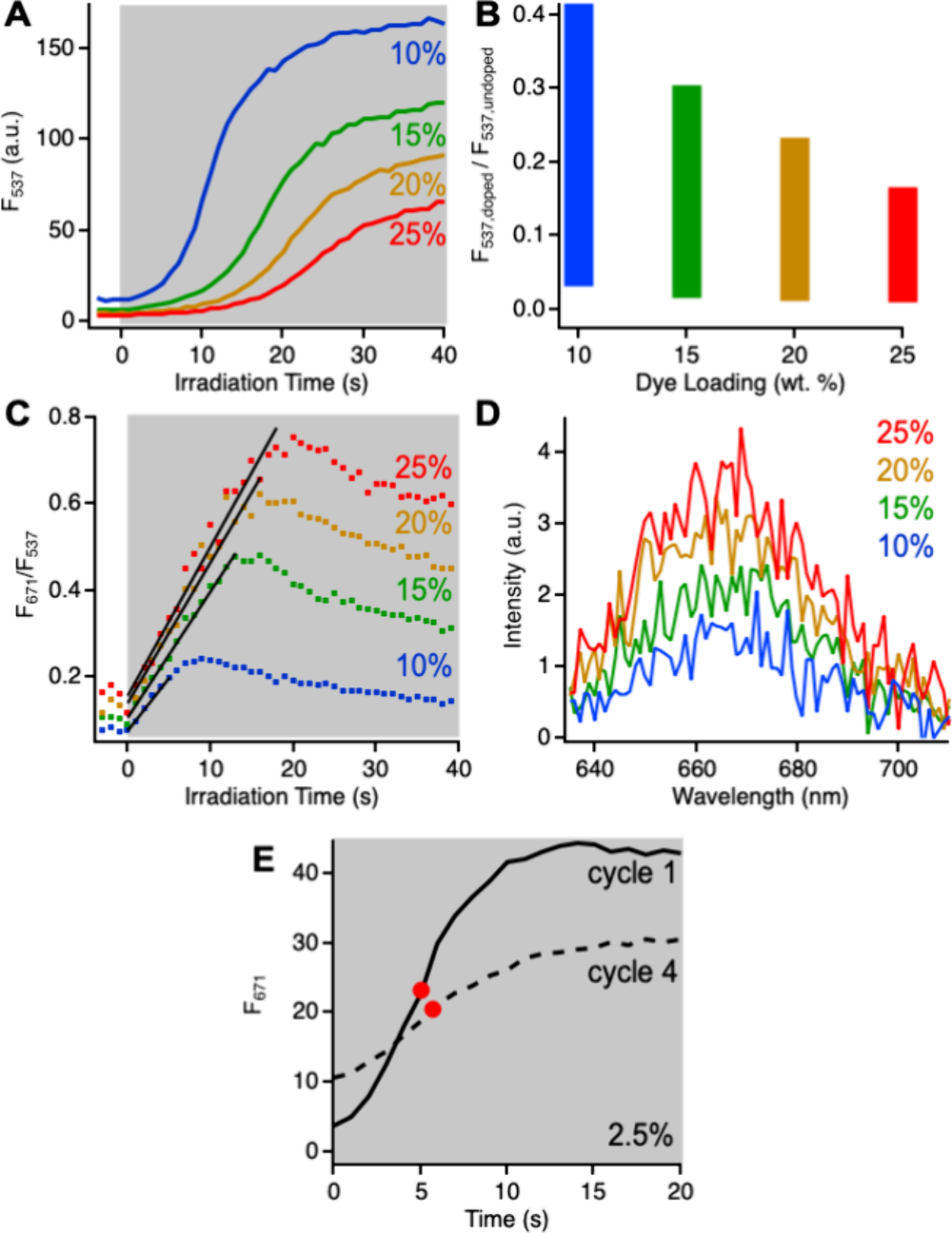
(A) Evolution of CPN fluorescence signal in doped CPNs during irradiation (shaded area, 0.87 mW/cm^2^) as a function of dye loading (wt %). (B) Dynamic range of CPN fluorescence intensities accessed during the experiment in (A). (C) Ratio of fluorescence intensities as a function of irradiation time and dye loading. (D) Fluorescence spectra upon direct excitation of Cy5 dyes. (E) Evolution of Cy5 fluorescence in doped CPNs during irradiation (0.87 mW/cm^2^) after the first and fourth cycles of dye addition. Dye amounts equivalent to 2.5 wt % were added to the sample for each dope-irradiate cycle. Red dots depict the point at which half the signal growth is complete.

We varied the composition of the dye-doped CPNs from 10 to 25 wt % at a constant irradiation intensity (0.87 mW/cm^2^). As the dye loading level increases, the initial fluorescence intensity decreases due to the presence of additional quenching HyCy5 dyes (Figure 9A). LED irradiation induces the same pattern of signal growth as observed in the irradiation intensity experiments (Figure 8A). Here, increasing the dye loading slows signal growth and shifts the region of maximum responsiveness to later times. The time at which the greatest rate of change in fluorescence intensity is observed ranges from 10 s at 10 wt % to 25 s at 25 wt %. With irradiation intensities held constant in this experiment, it is the dye loadings that are responsible for the kinetic differences. The addition of more HyCy5 quenchers appears to slow superoxide production, likely because HyCy5 quenching out-competes the intersystem crossing process the precedes superoxide generation (Figure 3B). The more HyCy5 quenchers that are present, the slower superoxide production becomes. HyCy5′s influence on superoxide generation means that the doped CPNs are likely reporting on substantially lower superoxide concentrations than those obtained from undoped CPNs in the Cyt C assay. Converting amplified fluorescence signals into superoxide concentrations will be a subject of future investigation.

These results demonstrate that both irradiation intensity and dye loading can be used to tune the rate of superoxide production and the fluorescence signal’s temporal evolution. When dye loading is manipulated, the F_537_ signals do not continue to grow to the same end point as they did when irradiation intensity was varied. Here, the final signal level decreases with increasing dye loading due to the presence of unreacted HyCy5 quenchers that suppress fluorescence intensities throughout the experiment (Figure 9A). The number of HyCy5 dyes that remain unreacted at the end of the experiment appears to increase as dye loading increases. Normalizing the initial and final fluorescence intensities (F_537_) for each sample to the fluorescence intensity of undoped CPNs demonstrates this point and enables visualization of the dynamic range of fluorescence changes (Figure 9B). The 25 wt % sample utilizes a small range of the possible fluorescence intensities, reaching only 17% of the undoped fluorescence intensity after 40 s irradiation. The dynamic range of fluorescence intensities increases as the dye loading decreases. No doped sample will reach the fluorescence intensity of the undoped CPNs because some of the CPN’s S_1_ excited states are deactivated by the FRET pathway to produce Cy5 fluorescence. Here, the 10 wt % CPNs reach just over 40% of the undoped fluorescence intensity.

The greater dynamic range of fluorescence intensities observed at lower dye loadings suggests that CPNs with fewer dye dopants are preferable to those with higher dye loadings. However, the ratiometric signals (F_671_/F_537_) exhibit a different trend. At all dye loadings studied, F_671_/F_537_ is initially linear before leveling out and declining as described previously (Figures 9C and 6C). The duration of the linear region increases with increasing dye loading due to the increased duration of the slow-growth region of the fluorescence intensity traces. The linear signal is observed for 5 s at 10 wt % up to 18 s at 25 wt %. These results demonstrate that dye loading can be used to tune the responsiveness of the fluorescence to superoxide. The larger dynamic range of fluorescence intensities that is possible with a low dye loading may be more appropriate for off-to-on fluorescence probing of superoxide. Alternatively, the extended linear signal observed in samples with a higher dye loading may be more suited to tracking gradual increases in superoxide concentration as a function of time.

To determine whether the composition-dependent responses reflect differences in the maximum amount of superoxide being detected, we irradiated the four samples until the fluorescence response reached a plateau (20–50 s) and then recorded the Cy5 fluorescence upon direct excitation. Directly exciting the dyes removes the confounding influences of HyCy5 quenching and amplified FRET on the fluorescence intensity, enabling us to determine whether the amount of superoxide-generated Cy5 dyes varies with composition. We observe that the fluorescence intensity of directly excited Cy5 increases slightly but reproducibly with dye loading (Figure 9D). The concentration of Cy5 dyes is too low to detect these differences by absorbance, which would facilitate a more quantitative analysis. Qualitatively, the fluorescence intensity differences suggest that more superoxide is trapped when more dyes are present, a feature that could be useful for tuning the sensitivity range of the system.

Dye loading clearly influences both the kinetics of superoxide production and the amount of superoxide that can be trapped and detected. Since the fluorescence time trajectories all exhibit a plateauing effect, we sought to determine whether dye loading also shuts down superoxide production once some number of Cy5 FRET acceptors have been generated. We investigated this question by adding additional HyCy5 dyes to a sample after it had been irradiated and reached the familiar plateau (Figure 9E). We used the Cy5 fluorescence signal for monitoring since it always plateaus at the end of a cycle. Irradiating the redoped CPNs produced the same familiar activation of fluorescence, albeit with some intensity differences due to the increased HyCy5 concentration. We repeated this process for 4 total cycles of doping and irradiation and observed fluorescence activation each time. If a competitive FRET process slows or shuts off superoxide production, we would expect to observe slower growth of the fluorescence signal as more Cy5 is produced in each subsequent cycle. In the case of a lightly doped sample (2.5 wt %), the fluorescence signal’s growth remained fairly constant, with the time at which half the signal growth is complete increasing from only 5.0 to 5.7 s from the first to fourth cycles. The time at which the maximum rate of change is observed was identical for both cycles at 5.0 s. These results suggest that activation of the FRET pathway does not shut down superoxide production. The plateauing of the fluorescence signal appears to reflect that all HyCy5 dyes that are able to react with superoxide have done so rather than that the CPNs’ ability to generate superoxide has been exhausted.

## Conclusions

Hydrocyanine-doped PFBT CPNs act as off-to-on fluorescence reporters of superoxide produced by the CPNs. Nonfluorescent HyCy5 is an efficient quencher of PFBT fluorescence in as-prepared CPNs. LED irradiation of HyCy5-doped CPNs in aerated aqueous suspension induces the formation of superoxide, which is detected by a traditional Cytochrome C assay and by the activation of fluorescence. This off-to-on fluorescence switching behavior is due to the reaction of the dye with superoxide, which converts quenching HyCy5 to fluorescent Cy5. This reaction step simultaneously removes a HyCy5 quencher from the system and produces a FRET-accepting Cy5 dye, and the net effect is the activation of emission from both PFBT (yellow-green) and Cy5 (red) channels. HyCy5 has previously been shown by others to act as a fluorescent superoxide reporter on its own. When doped onto the CPNs and excited via FRET in the present work, the Cy5 emission intensity is nearly 50 times greater than when the dyes are excited directly. The signal amplification is potentially even larger: the correlated nature of the PFBT and Cy5 emissions means that the larger PFBT signal can also be used as a readout, increasing the antenna effect to more than 100 in samples with 15 wt % dye loading. Alternatively, the two signals can be used as a ratiometric readout that is linear at early irradiation times.

The PFBT CPNs studied here both produce superoxide and act as fluorescence reporters, and the dye dopants that facilitate reporting also influence superoxide generation. Our data indicate that dye loading levels govern the initial and final fluorescence intensities as well as set the upper limit on the amount of superoxide that generates a fluorescence response. CPN dye loading levels affect not only these properties but also the rate of superoxide production, which slows as HyCy5 loading levels increase. The intertwined nature of superoxide production and reporting functions suggests that it could be beneficial to separate them by identifying CPNs that do not produce superoxide for future fluorescence reporting studies. Alternatively, this interplay could be used to control the rate of superoxide production for future release and report applications.
